# A List of the Plants Growing Spontaneously in the Vicinity of Quincy, Florida

**Published:** 1845-06

**Authors:** A. W. Chapman


### WESTERN JOURNAL

OF

### MEDICINE AND SURGERY.

JUNE, 1845.

### ART. I.—*A List of the Plants growing spontaneously in the vicinity of Quincy, Florida.* By A. W. CHAPMAN, M.D.

RANUNCULACEÆ.

Clematis cylindrica, *Sims.*
reticulata, *Walt.*
viorna, *L.*
Virginiana, *L.*
Thalictrum anemonoides, *Mx.*
Cornuti, *L.*
Hepatica triloba, *Chaix.*
Isopyrum biternatum, *T.&G.*
Ranunculus acris, *L.*
abortivus, *L.*
parviflorus, *L.*
palmatus, *Ell.*
Ranunculus repens, *L.*
recurvatus, *L.*
Aquilegia Canadensis, *L.*
Delphinium azureum, *Mx.*
Xanthoriza apiifolia, *L'Herit.*

PAPAVERACEÆ.

Sanguinaria Canadensis, *L.*
Argemone Mexicana, *L.*
b. albiflora, *D.C.*

MAGNOLIACEÆ.

Magnolia glauca, *L.*
grandiflora, *L.*
macrophylla, *Mx.*
Frazeri, *Walt.*
b. pyramidata, *Nut.*
Liriodendron Tulipifera, *L.*
Illicium Floridanum, *Ellis.*

ANONACEÆ.

Uvaria parviflora, *T.&G.*

obovata, *T.&G.*

triloba, *T.&G.*

MENISPERMACEÆ.

Cocculus Carolinus, *D.C.*
Menispermum Lyoni, *Pursh.*

BERBERIDACEÆ.

Podophyllum peltatum, *L.*
Croomia pauciflora, *Torr.*

CABOMBACEÆ.

Cabomba Caroliniana, *Gray.*
Brasenia peltata, *Pursh.*

NELUMBIACEÆ.

Nelumbium luteum, *Willd.*

NYMPHÆACEÆ.

Nymphæa odorata, *Ait.*

Nuphar advena, *Ait.*

SARRACENIACEÆ.

Sarracenia flava, *L.*
purpurea, *L.*
Drummondii, *Cr'm.*
Psittacina, *Mx.*
variolaris, *Mx.*

FUMARIACEÆ.

Corydalis aurea, *Willd.*
Fumaria officinalis, *L.*

CRUCIFERÆ.

Nasturtium limosum, *Nutt.*
natans, *D.C.*
v. brevistylum, *T.*
Cardamine rotundifolia, *Mx.*
hirsuta, *L.*
Ludoviciana, *Hook.*
Dentaria, laciniata, *Muhl.*
Sisymbrium canescens, *Nutt.*

Warea cuneifolia, *Nutt.*
amplexifolia, *Nutt.*
Senebiera pinnatifida, *D.C.*
Lepidium Virginicum, *L.*

POLYGALACEÆ.

Polygala sanguinea, *L.*
cruciata, *L.*
lutea, *L.*
nana, *D.C.*
Baldwinii, *Nutt.*
incarnata, *L.*
setacea, *Mx.*
Boykinii, *Nutt.*
Chapmanii, *T.&G.*
polygama, *Walt.*
grandiflora, *Walt.*
corymbosa, *Mx.*
attenuata, *Nutt.*
Hookeri, *T.&G.*

VIOLACEÆ.

Viola palmata, *L.*
cucullata, *Ait.*
villosa, *Walt.*
primulæfolia, *L.*
lanceolata, *L.*
Muhlenbergii, *Torr.*
v. multicaulis, *T.&G.*
hastata, *Mx.* ?

DROSERACEÆ.

Drosera brevifolia, *Pursh.*
rotundifolia, *L.*
filiformis, *Raf.*
Parnassia Caroliniana, *Mx.*

CISTACEÆ.

Helianthemum corymbosum, *M.*
Carolinianum, *M.*
Lechea major, *Mx.*
minor, *Lam.*

HYPERICACEÆ.

Ascyrum crux-andreæ, *L.*
pumilum, *Mx.*
stans, *Mx.*
v. obovatum, *Chap.*
microsepalum, *T&G*
Hypericum galioides, *Lam.*
fasciculatum, *Lam.*
maculatum, *Walt.*
myrtifolium, *Lam.*
ambiguum, *Ell.*
nudiflorum, *Mx.*
cistifolium, *Lam.*
pilosum, *Walt.*
angulosum, *Mx.*
mutilum, *L.*
Sarothra, *Mx.*
Elodea petiolata, *Pursh.*
Virginica, *Nutt.*

ILLECEBRACEÆ.

Anychia Baldwinii, *T.&G.*

Siphonychia Americana, *T&G*

Stipulicida setacea, *Mx.*

Spergula rubra, *T.&G.*

CARYOPHYLLACEÆ.

Mollugo verticillata, *L.*
Sagina decumbens, *T.&G.*
Arenaria squarrosa, *Mx.*
serpyllifolia, *L.*
Stellaria media, *Smith.*
lanuginosa, *T.&G.*
Cerastium vulgatum, *L.*
viscosum, *L.*
Silene Baldwinii, *Nutt.*
Antirrhina, *L.*

POTULACACEÆ.

Portulaca oleracea, *L.*

LINACEÆ.

Linum Virginianum, *L.*

GERANIACEÆ.

Geranium Carolinianum, *L.*

BALSAMINACEÆ.

Impatiens pallida, *Nutt.*

OXALIDACEÆ.

Oxalis violacea, *L.*
corniculata, *L.*
stricta, *L.*

XANTHOXYLACEÆ.

Xanthoxylum Carolinianum, *L.*
Ptelea trifoliata, *L.*

ANACARDIACEÆ.

Rhus glabra, *L.*
copallina, *L.*
venenata, *D.C.*
Toxicodendron, *L.*
aromatica, *Ait.*

TERNSTRÆMIACEÆ.

Gordonia Lasianthus, *L.*
Stuartia Malachodendron, *L.*

MALVACEÆ.

Malva papaver, *Cav.*
Modiola multifida, *Mœnch.*

Sida spinosa, *L.*
Elliottii, *T.&G.*
rhombifolia, *L.*
Hibiscus aculeatus, *Walt.*
incanus, *Wendl.*

TILIACEÆ.

Tilia pubescens, *Ait.*

VITACEÆ.

Vitis bipinnata, *T.&G.*

indivisa, *Willd.*

æstivalis, *Mx.*

cordifolia, *Mx.*

vulpina, *L.*

Ampelopsis quinquefolia, *Mx.*

ACERACEÆ.

Acer dasycarpum, *Ehrh.*
rubrum, *L.*
nigrum, *Mx.*
Negundo aceroides, *Mœnch.*

HYPPOCASTANACEÆ.

Æsculus Pavia, *L.*

CELASTRACEÆ.

Euonymus atropurpureus, *Jac.*
Americanus, *L.*

RHAMNACEÆ.

Berchemia volubilis, *D.C.*
Rhamnus Carolinianus, *Walt.*
Sageretia Michauxii, *Brogn.*
Ceanothus Americanus, *L.*
microphyllus, *Mx.*

LEGUMINOSÆ.

Vicia Caroliniana, *Walt.*
micrantha, *Nutt.*
acutifolia.
Phaseolus perennis, *Walt.*
sinuatus, *Nutt.*
diversifolius, *Pers.*
helvolus, *L.*
Erythina herbacea, *L.*
Apios tuberosa, *Mœnch.*
Wistaria frutescens, *D.C.*
Rhynchosia tomentosa, *T.&G.*
Pitcheria galactoides, *Nutt.*
Galactia glabella, *Mx.*
pilosa, *Nutt.*
mollis, *Mx.*
brachypoda, *T.&G.*
sessiliflora, *T.&.G.*
Clitoria Mariana, *L.*
Centrosema Virginiana, *Benth.*
Amphicarpæa monoica, *T.&G.*

Sesbania macrocarpa, *Muhl.*
Glottidium Floridanum, *D.C.*
Tephrosia Virginiana, *Pers.*
spicata, *T.&G.*
hispidula, *Pursh.*
chrysophylla, *Pursh.*
Indigofera Caroliniana, *Walt.*
Psoralea canescens, *Mx.*
melilotoides, *Mx.*
b. *T.&G.*
Amorpha fruticosa, *L.*
Caroliniana, *Croom.*
Petalostemum carneum, *Mx.*
gracile, *Nutt.*
corymbosum, *Mx.*
Trifolium Carolinianum, *Mx.*
reflexum, *L.*
Astragalus obcordatus, *Ell.*
Phaca villosa, *Nutt.*
Zornia tetraphylla, *Mx.*
Stylosanthes elatior, *Swartz.*
Æschynomene viscidula, *Mx.*
Desmodium nudiflorum, *D.C.*
acuminatum, *D.C.*
canescens, *D.C.*
Dillenii, *Darl.*
cuspidatum, *T.&G*
viridiflorum, *Beck.*
rhombifolium, *D.C.*
Marilandicum, *B't.*
ciliare, *D.C.*
rigidum, *D.C.*
strictum, *D.C.*
paniculatum, *D.C.*
rotundifolium, *D.C.*
lineatum, *D.C.*
Lespedeza procumbens, *Mx.*
Lespedeza repens, *T.&G.*
violacea, *Pers.*
hirta, *Ell.*
capitata, *Mx.*
Crotalaria ovalis, *Pursh.*
Purshii, *D.C.*
Lupinus perennis, *L.*
v. *T.&G.*
villosus, *Willd.*
Baptisia lanceolata, *Ell.*
megacarpa, *Chap.*
simplicifolia, *Croom.*
alba, *Br.*
leucantha, *T.&G.*
Lecontii, *T.&G.*
Circis Canadensis, *L.*
Cassia occidentalis, *L.*
obtusifolia *L.*
Marilandica, *L.*
Chamæcrista, *L.*
nictitans, *L.*
Gleditschia triacanthos, *L.*
monosperma, *Walt*
Schrankia angustata, *T.&G.*

ROSACEÆ.

Chrysobalanus oblongifolius, *M*
Prunus Americana, *Marsh.*
Chicasa, *Mx.*
Cerasus serotina, *D.C.*
Caroliniana, *Mx.*
Spiræa opulifolia, *L.*
Agrimonia Eupatoria, *L.*
parviflora, *Ait.*
Fragaria Virginiana, *Ehrh.*

Rubus villosus, *Ait.*
trivialis, *Mx.*
cuneifolius, *Pursh.*
Rosa setigera, *Mx.*
lucida, *Ehrh.*
lævigata, *Mx.*
Cratægus coccinea, v. *L.*
apiifolia, *Mx.*
spathulata, *Mx.*
æstivalis, *T. & G.*
parvifolia, *Ait.*
Pyrus angustifolia, *Ait.*
arbutifolia, *L.*
Amelanchier Canadensis, *T. & G.*

CALYCANTHACEÆ.

Calycanthus Floridus, *L.*

MELASTOMACEÆ.

Rhexia Mariana, *L.*
lanceolata, *Walt.*
stricta, *Pursh.*
glabella, *Mx.*
ciliosa, *Walt.*
lutea, *Walt.*

LYTHRACEÆ.

Ammannia humilis, *Mx.*
Lythrum alatum, *Pursh.*
Decodon verticillatum, *Ell.*

ONAGRACEÆ.

Œnothera linearis, *Mx.*
biennis, *L.*
sinuata, *L.*
Gaura filipes, *Spach.*
Jussiæa decurrens, *D.C.*
leptocarpa, *Nutt.*
Ludwigia alternifolia, *L.*
hirtella, *Raf.*
virgata, *Mx.*
linearis, *Walt.*
linifolia, *Poir.*
cylindrica, *Ell.*
pilosa, *Walt.*
alata, *Ell.*
sphærocarpa, *Ell.*
microcarpa, *Mx.*
capitata, *Mx.*
palustris, *Ell.*
natans, *Ell.*
spathulata, *T. & G.*
arcuata, *Walt.*

HALORAGEÆ.

Proserpinaca palustris, *L.*
pectinacea, *Lam.*
Myriophyllum verticillatum, *L.*
heterophyllum, *Mx.*
scabratum, *Mx.*

TURNERACEÆ.

Turnera cistoides, *L.*

PASSIFLORACEÆ.

Passiflora lutea, *L.*
incarnata, *L.*

CUCURBITACEÆ.

Melothria pendula, *L.*
Sicyos augulatus, *L.*

CACTACEÆ.

Opuntia vulgaris, *Mill.*

CRASSULACEÆ.

Penthorum sedoides, *L.*

SAXIFRAGACEÆ.

Hydrangia quercifolia, *Bartr.*
arborescens, *L.*
Decumaria barbara, *L.*
Philadelphus grandiflorus, *Willd.*

HAMAMELACEÆ.

Hamamelis Virginiana, *L.*

UMBELLIFERÆ.

Hydrocotyle interrupta, *Muhl.*
umbellata, *L.*
Hydrocotyle repanda, *Pers.*
ranunculoides, *L.*
Sanicula Marilandica, *L.*
Eryngium aquaticum, *L.*
virgatum, *Lam.*
Virginianum, *L.*
Baldwinii, *Spreng.*
Discopleura capillacea, *D.C.*
Leptocaulis divaricatus, *D.C.*
Cicuta maculata, *L.*
Sium lineare, *Mx.*
Neurophyllum longifolium, *T.&G.*
Thaspium cordatum, *T. & G.*
barbinode, *Nutt.*
Archangelica hirsuta, *T. & G.*
dentata, *Chap.*
Piedmannia teretifolia, *D.C.*
Archemora rigida, *D.C.*
Daucus pusillus, *Mx.*

ARALIACEÆ.

Aralia spinosa, *L.*

CORNACEÆ.

Cornus stricta, *Lam.*
asperifolia, *Mx.*
sericea, *L.*
florida, *L.*

LORANTHACEÆ.

Viscum flavescens, *Pursh.*

CAPRIFOLIACEÆ.

Lonicera sempervirens, *Ait.*
Sambucus Canadensis, *L.*
Viburnum nudum, *L.*
prunifolium *L.*
obovatum, *Walt.*
dentatum, *L.*
b. *T. & G.*
acerifolium, *L.*

RUBIACEÆ.

Galium hispidulum, *Mx.*
uniflorum, *Mx.*
trifidum, *L.*
pilosum, *Ait.*
circæzans, *Mx.*
Spermacoce glabra, *Mx.*
Chapmanii, *T. & G.*
Diodia Virginiana, *L.*
teres, *Walt.*
Cephalanthus occidentalis, *L.*
Mitchella repens, *L.*
Pinckneya pubens, *Mx.*
Hedyotis rotundifolia, *T. & G.*
purpurea, *T. & G.*
stenophylla, *T. & G.*
Boscii, *D.C.*
glomerata, *Ell.*
Mitreola petiolata, *T. & G.*
sessilifolia, *T. & G.*
Polypremum procumbens, *L.*

VALERIANACEÆ.

Fedia radiata, *Mx.*

COMPOSITÆ.

Vernonia augustifolia, *Mx.*
fasciculata, *Mx.*
ovalifolia, *T. & G.*
Elephantopus tomentosus, *L.*
Carolinianus, *Willd.*
Sclerolepis verticillata, *Cass.*
Carphephorus pseudo-Liatris, *Cass.*
Liatris elegans, *Willd.*
squarrosa, *Willd.*
tenuifolia, *Nutt.*
gracilis, *Pursh.*
graminifolia, *Willd.*
spicata, *Willd.*
scariosa, *Willd.*
odoratissima, *Willd.*
paniculata, *Willd.*
Chapmanii, *T. & G.*
Kuhnia Eupatorioides, *L.*
Brickellia cordifolia, *Ell.*
Eupatorium purpureum, *L.*
fæniculaceum, *Willd.*
coronopifolium, *Wil.*
pinnatifidum, *Ell.*
hyssopifolium, *L.*
leucolepis, *T. & G.*
parviflorum, *Ell.*
album, *L.*
teucrifolium, *Willd.*
rotundifolium, *L.*
perfoliatum, *L.*
serotinum, *Mx.*
aromaticum, *L.*
incarnatum, *Walt.*
Mikania scandens, *Willd.*
Cenoclinum cælestinum, *D.C.*
Seriocarpus tortifoluis, *Nees.*
Aster concolor, *L.*

Aster adnatus, *Nutt.*
patens, *Ait.*
undulatus, *L.*
ericoides, *L.*
dumosus, *L.*
miser, *L.*
simplex, *Willd.*
Chapmanii, *T. & G.*
divaricatus, *Nutt.*
Eryngiifolius, *T. & G.*
Erigeron Canadense, *L.*
Philadelphicum, *L.*
strigosum, *L.*
vernum, *T. & G.*
Diplopappus linearifolius, *Hook.*
amygdalinus, *T. & G.*
obovatus, *T. & G.*
Boltonia diffusa, *Ell.*
Solidago discoidea, *T. & G.*
cæsia, *L.*
virgata, *Mx.*
puberula, *Nutt.*
petiolaris, *Ait.*
speciosa, *Nutt.*
sempervirens, *L.*
angustifolia, *Ell.*
patula, *Muhl.*
arguta, *Ait.*
gracillima, *T. & G.*
altissima, *L.*
amplexicaulis, *T. & G.*
brachyphylla, *Chap.*
pilosa, *Walt.*
odora, *Ait.*
tortifolia, *Ell.*
nemoralis, *Ait.*
Leavenworthii, *T. & G.*
Canadensis, *L.*
pauciflosculosa, *Mx.*

Solidago tenuifolia, *Pursh.*
Bigelowia nudata, *D.C.*
Heterotheca scabra, *D.C.*
Chrysopsis graminifolia, *Nutt.*
oligantha, *Chap.*
Mariana, *Nutt.*
trichophylla, *Nutt.*
hyssopifolia, *Nutt.*
gossypina, *Nutt.*
Baccharis halimifolia, *L.*
angustifolia, *Mx.*
Pluchea bifrons, *D.C.*
camphorata, *D.C.*
Pterocaulon pycnostachyum, *Ell.*
Borrichia frutescens, *D.C.*
Eclypta erecta, *L.*
Polymnia Uvedalia, *L.*
Chrysogonum Virginianum, *L.*
Silphium compositum, *Mx.*
Asteriscum, *L.*
Berlandiera tomentosa, *T. & G.*
Iva frutescens, *L.*
microcephala, *Nutt.*
Ambrosia trifida, *L.*
artemesiæfolia, *L.*
Xanthium strumarium, *L.*
Melanthera hastata, *Mx.*
Zinnia multiflora, *L.*
Heliopsis lævis, *Pers.*
Tetragonotheca helianthoides, *L.*
Echinacea atrorubens, *Nutt.*
Rudbeckia hirta, *L.*
fulgida, *Ait.*
triloba, *L.*
laciniata, *L.*
heterophylla, *T. & G.*
Lepachys pinnata, *T. & G.*
Helianthus augustifolius, *L.*
Radula, *T. & G.*

Helianthus heterophyllus, *Nutt.*
tomentosus, *Mx.*
strumosus, *L.*
divaricatus, *L.*
microcephalus, *T.&G.*
Helianthella tenuifolia *T.&G.*
Actinomeris squarrosa, *Nutt.*
nudicaulis, *Nutt.*
Coreopsis aurea, *Ait.*
tripteris, *L.*
senifolia, *Mx.*
auriculata, *L.*
lanceolata, *L.*
gladiata, *Walt.*
angustifolia, *Ait.*
integrifolia, *Poir,*
nudata, *Nutt.*
Bidens frondosa, *L.*
chrysanthemoides, *Mx.*
bipinnata, *L.*
Verbesina Virginica, *L.*
Gaillardia lanceolata, *Mx.*
Palafoxia integrifolia, *T.&G.*
Hymenopappus scabiosæus, *L'H.*
Helenium autumnale, *L.*
quadridentatum, *Ell.*
Leptopoda Helenium, *Nutt.*
puberula, *Macb.*
Baldwinia uniflora, *Nutt.*
Actinospermum angustifolium, *Ell.*
Marshallia angustifolia, *Pursh.*
lanceolata, *Pursh.*
Leucanthemum vulgare, *Lam.*
Artemesia caudata, *Mx.*
Gnaphalium polycephalum, *Mx.*
Antennaria plantaginifolia, *Hook.*
Cacalia diversifolia, *T.&G.*
ovata, *Walt.*
Senecio lobatus, *Pers.*
aureus, *L.*
Elliottii, *T.&G.*
tomentosus, *Mx.*
Arnica nudicaulis, *Ell.*
Cirsium Lecontii, *T.&G.*
repandum, *Mx.*
horridulum, *Mx.*
Chaptalia tomentosa, *Vent.*
Apogon humilis, *Ell.*
Krigia Virginica, *Willd.*
Cynthia Dandelion, *D.C.*
Hieracium Gonovii, *L.*
Lygodesmia aphylla, *D.C.*
Pyrrhopappus Carolinianus, *D.C.*
Lactuca graminifolia, *Mx.*
elongata, *Muhl.*
Mulgedium Floridanum, *D.C.*

LOBELIACEÆ.

Lobelia Cardinalis, *L.*
amæna, *Mx.*
glaudulosa, *Walt.*
crassiuscula, *Mx.*
paludosa, *Nutt.*
puberula, *Mx.*

CAMPANULACEÆ.

Campanula Americana, *L.*
amplexicaulis, *Mx.*

ERICACEÆ.

Andromeda axillaris, *Ait.*

Andromeda arborea, *L.*
frondosa, *Muhl.*
Mariana, *L.*
nitida, *Walt.*
paniculata, *Pursh.*
racemosa, *Mx.*
rigida, *Pursh.*
phyllicrifolia, *Hook.*
Epigæa repens, *L.*
Mylocarium ligustrinum, *Willd.*
Clethra tomentosa. *Lam.*
alnifolia, *L.*
Cyrilla racemiflora. *Walt.*
Kalmia hirsuta, *Walt.*
latifolia, *L.*
Rhododendron viscosum, *T.*
nudiflorum, *T.*
punctatum, *Ell.*
Vaccinium arboreum, *Marsh.*
corymbosum, *L.*
dumosum, *Curtis.*
hirtellum, *Ait.*
frondosum, *L.*
stamineum, *L.*
myrsinites, *Mx.*
myrtilloides, *Ell. Mx.*?
galezans, *Mx.*
Monotropa uniflora, *L.*
lanuginosa, *Mx.*

AQUIFOLIACEÆ.

Ilex opaca, *Ait.*
myrtifolia, *Walt.*
vomitoria? *Ait.*

EBENACEÆ.

Diospyros Virginiana, *L.*
Styrax grandifolium, *Ait.*
glabrum, *Ell.*
pulverulentum, *Mx.*
Symplocos tinctoria, *L'Herit.*

SAPOTACEÆ.

Bumelia lanuginosa, *Mx.*
reclinata, *Ph.*
tenax, *Willd.*

PRIMULACEÆ.

Samolus Valerandi, *L.*
ebracteatus, *Kunth.*
Lysimachia ciliata, *L.*
sp. nov.
Micranthemum orbiculatum, *Mx.*

PLANTAGINACEÆ.

Plantago pusilla, *Nutt.*

Virginica, *L.*

PLUMBAGINACEÆ.

Statice Caroliniana, *Walt.*

LENTIBULACEÆ.

Pinguicula elatior, *Mx.*

Pinguicula lutea, *Walt.*
pumila, *Mx.*
Utricularia inflata, *Walt.*
setacea, *Mx.*
bipartita, *Ell.*
personata, *Lecont.*
purpurea, *Walt.*

OROBANCHACEÆ.

Orobanche Americana, *L.*
uniflora, *L.*
Epiphegus Americanus, *Nutt.*

BIGNONIACEÆ.

Bignonia radicans, *L.*
capreolata, *L.*
Catalpa cordifolia, *Duham.*

ACANTHACEÆ.

Justicia ensiformis, *Walt.*
brachiata, *Pursh.*
sp. nov.
Ruellia strepens, *L.*
oblongifolia, *Mx.*
tubiflora, *Lecont.*
humistrata, *Mx.*
Elytraria virgata, *Mx.*

SCROPHULARIACEÆ.

Scrophularia Marilandica, *L.*
Antirrhinum Canadense, *L.*
Antirrhinum sp. nov.
Chelone glabra, *L.*, var.
Gerardia quercifolia, *Pursh.*
Pedicularia, *L.*
linifolia, *Nutt.*
purpurea, *L.*
tenuifolia, *Vahl.*
aphylla, *Nutt.*
fasciculata, *Ell.*
filifolia? *Nutt.*
setacea, *Nutt.*
stenopoda, sp. nov.
Gratiola pilosa, *Mx.*
acuminata, *Walt.*
sphærocarpa, *Ell.*
quadridentata, *Mx.*
Virginica, *L.*
Mimulus alatus, *Ait.*
ringens, *L.*
Pentstemon pubescens, *Ait.*
lævigatus, *Ait.*
Veronica peregrina, *L.*
Verbascum Thapsus, *L.*
Blattaria, *L.*
Buchnera Americana, *L.*
Siphonostegia, sp., *Benth.*
Herpestis amplexifolia, *Pursh.*
cuneifolia, *Pursh.*
Schwalbea Americana, *L.*
Lindernia attenuata, *Muhl.*
Seymeria tenuifolia, *Pursh.*
pectinata, *Pursh.*
Macranthera Lecontii, *Torr.*

VERBENACEÆ.

Verbena angustifolia, *Mx.*
Caroliniana, *L.*

Verbena urticifolia.
Aubletia.
Callicarpa Americana, *L.*
Phryma leptostachya, *L.*
Zapania nodiflora, *Pursh.*

LABIATÆ.

Hyptis radiata, *L.*
Lamium amplexicaule, *L.*
Lycopus Virginicus, *L.*
angustifolius, *Ell.*
Monarda punctata, *L.*
Physostegia variegata, *Pursh.*
Prunella vulgaris, *L.*
Pycnanthemum incanum, *Mx.*
hyssopifolium, *B.*
nudum, *Nutt.*
linifolium, *Pursh.*
Salvia azurea, *L.*
lyrata, *L.*
urticifolia, *L.*
Scutellaria integrifolia, *L.*
parvula, *Mx.*
pilosa, *Mx.*
ovalifolia, *Pers.?*
Stachys hispida, *Pursh.*
Teucrium Canadense, *L.*
Trichostema dichotoma, *L.*
Collinsonia anisata, *Mx.*
punctata, *Ell.*
Ceranthera linearifolia, *Ell.*
Micromeria Brownei, *Benth.*
Gardoquia Hookeri, *Benth.*
Keithia, sp., *Benth.*

BORAGINACEÆ.

Onosmodium hispidum, *Mx.*
Batschia Gmellini, *Mx.*
Cynoglossum Virginicum, *L.*
Myosotis palustris, *Roth.*
Lithospermum, sp. nov.
Heliotropium Indicum, *L.*

HYDROLEACEÆ.

Hydrolea quadrivalvis, *Walt.*

POLEMONIACEÆ.

Phlox aristata, *Mx.*
maculata, *L.*
pilosa, *L.*
divaricata, *L.*
subulata, *L.*

CONVOLVULACEÆ.

Convolvulus panduratus, *L.*
tenellus, *Lam.*
aquaticus, *Walt.*
repens, *L.*
sagittæfolius, *Mx.*
tamnifolius. *Mx.*
trichocarpus, *Mx.*
Dichondra Caroliniensis, *Mx.*
Evolvulus sericeus, *Mx.*
Cuscuta Americana, *L.*

SOLANACEÆ.

Solanum nigrum, *L.*
Carolinense, *L.*
Physalis lanceolata, *Mx.*
angulata, *Walt.*
Datura stramonium, *L.*
Lycium Carolinianum, *Walt.*
Atropa physaloides, *Willd.*

GENTIANACEÆ.

Gentiana Catesbæi, *Walt.*
ochroleuca, *Willd.*
angustifolia, *Mx.*
Centaurella verna, *Mx.*
paniculata, *Mx.*
Villarsia trachysperma, *Ell.*
Sabbatia chloroides, *Pursh.*
brachiata, *Ell.*
angularis, *Pursh.*
paniculata. *Pursh.*
gentianoides, *Ell.*
corymbosa, *Bald.*
calycosa, *Pursh.*
sp. nov.

SPIGELIACEÆ.

Spigelia Marilandica, *L.*
gentianoides, sp. nov.

APOCYNACEÆ.

Apocynum pubescens, *Br.*
Amsonia angustifolia, *Mx.*
Amsonia latifolia, *Mx.*
Gelsemium nitidum, *Mx.*
Echites difformis, *Walt.*

ASCLEPIADACEÆ.

Asclepias amplexicaulis, *Mx.*
cinerea, *Walt.*
obtusifolia, *Mx.*
paupercula, *Mx.*
tuberosa, *L.*
variegata, *L.*
verticillata, *L.*
angustifolia, *Ell.*
obovata, *Ell.*
parviflora, *L.*
connivens, *Bald.*
longifolia, *Mx.*
viridis, *Walt.*
viridiflora, *Pursh.*
linifolia, sp. nov.
Gonolobus macrophyllus, *Mx.*
hirsutus, *Mx.*
Lyonia maritima, *Ell.*
Podostigma pubescens, *Ell.*

OLEACEÆ.

Olea Americana, *L.*
Chionanthus Virginica, *L.*
Fraxinus platycarpa, *Mx.*

ARISTOLOCHIACEÆ.

Aristolochia serpentaria, *L.*
tomentosa, *Nutt.*

Asarum arifolium, *Mx.*

CHENOPODIACEÆ.

Chenopodium ambrosioides, *L.*
anthelminticum, *L.*
Salicornia ambigua, *Mx.*

AMARANTHACEÆ.

Amaranthus spinosus, *L.*
pumilus, *Ell.?*
hybridus, *Willd.*
albus, *Willd.*
Oplotheca Floridana, *Nutt.*

NYCTAGINACEÆ.

Boerhaavia erecta, *Wild.*

POLYGONACEÆ.

Polygonum Pennsylvanicum, *L.*
punctatum, *Ell.*
Virginianum, *L.*
Convolvulus, *L.*
hirsutum, *Mx.*
incarnatum, *Ell.*
setaceum, *Baldwin.*
gracile, *Nutt.*
sp. nov.
Eriogonum tomentosum, *Mx.*
Brunnichia cirrhosa, *Mx.*
Rumex crispus, *L.*
Rumex verticillatus, *L.*
acetosella, *L.*

PHYTOLACCACEÆ.

Phytolacca decandra, *L.*

LAURACEÆ.

Laurus Benzoin, *L.*
Carolinensis, *Catesb.*
geniculata, *Walt.*
melissæfolia, *Walt.*
Sassafras, *L.*

SANTALACEÆ.

Nyssa biflora, *Walt.*
capitata, *Walt.*
multiflora, *Walt.*
tomentosa, *Mx.*

ULMACEÆ.

Ulmus alata, *Mx.*
Americana, *L.*
fulva, *Mx.*
racemosa, *Thomas.*
Celtis pumila, *Pursh.*
Planera aquatica, *Walt.*

SAURURACEÆ.

Saururus cernuus, *L.*

EUPHORBIACEÆ.

Euphorbia corollata, *L.*
cordifolia, *Ell.*
hypericifolia, *L.*
Ipecacuanha, *L.*
Peplus? *L.*
polygonifolia, *L.*
sp. nov.
Crotonopsis linearis, *Mx.*
Croton ellipticum, *Ell.*
glandulosum.
argyranthemum, *Mx.*
maritimum, *Walt.*
Phyllanthus obovatus, *L.*
Pachysandra procumbens, *Mx.*
Styllingia ligustrina, *Mx.*
sylvatica, *L.*
Tragia linearifolia, *Ell.*
urens, *L.*
urticifolia, *Mx.*
Jatropa stimulosa, *Mx.*
Acalypha Virginica, *L.*

EMPETRACEÆ.

Ceratiola ericoides, *Mx.*

JUGLANDACEÆ.

Carya aquatica, *Nutt.*
tomentosa, *Nutt.*
amara, *Mx.*
porcina, *Nutt.*
Juglans nigra, *L.*

CUPULIFERÆ.

Castanea pumila, *Mx.*
nana, *Muhl.*
Corylus Americanus, *Walt.*
Fagus sylvatica, *L.*
Quercus alba, *L.*
aquatica, *Nutt.*
Catesbæi, *Mx.*
cinerea, *Mx.*
coccinea, *Wang.*
falcata, *Mx.*
lyrata, *Walt.*
nigra, *L.*
obtusiloba, *Mx.*
Phellos, *L.*
Prinos, *L.*
rubra, *L.*
virens, *Ait.*
Castanea, *Willd.*
pumila, *Walt.*
Michauxii, *Nutt.*
maritima, *Willd.*

MYRICACEÆ.

Myrica Torreyana, sp. nov.
cerifera, *L.*
Carolinensis, *Wang.*

BETULACEÆ.

Ostrya Virginica, *Willd.*
Betula nigra, *L.*
Alnus serrulata, *Willd.*
Carpinus Americana, *Mx.*

SALICACEÆ.

Salix nigra, *Ell. L?*
Muhlenbergiana, *Willd.*
Populus angulata, *Ait.*

BALSAMIFLUÆ.

Liquidambar styraciflua, *L.*

PLATANACEÆ.

Platanus occidentalis, *L.*

URTICACEÆ.

Morus rubra, *L.*
Urtica Canadensis, *L.*
pumila, *L.*
Bœhmeria cylindrica, *L.*
Parietaria Pennsylvanica, *Muhl.*

CONIFERÆ.

Pinus australis, *Mx.*
inops, *Ait.*
tæda, *L.*
variabilis, *Lamb.*
Cupressus thyoides, *L.*
Juniperus Virginiana, *L.*
Taxodium distichum, *Rich.*
Torreya taxifolia, *Arnot.*
Taxus Croomii, sp. nov.

PALMÆ.

Chamærops Palmetto, *L.*
serrulata, *Pursh.*
hystrix, *Swartz.*
Sabal pumila, *Walt.*

ARACEÆ.

Orontium aquaticum, *L.*
Arum dracontium, *L.*
triphyllum, *L.*
Virginicum, *L.*

TYPHACEÆ.

Typha latifolia, *L.*
Sparganium Americanum? *Nutt.*

NAIADACEÆ.

Potamogeton natans, *L.*
fluitans, *L.*
setaceum, *Pursh.*
Zostera marina, *L.*

ALISMACEÆ.

Sagittaria graminea, *Mx.*
lancifolia *L.*
natans, *L.*
sagittæfolia, *Walt.*
pusilla, *Nutt.*

HYDROCHARIDACEÆ.

Hydrocharis spongiosa, *Bosc.*
Vallisneria spiralis, *Torr.*

BURMANNIACEÆ.

Tripterella capitata, *Mx.*
caerulea, *Mx.*
Apteria setacea, *Nutt.*

ORCHIDACEÆ.

Goodyera pubescens, *Willd.*
Cranichis multiflora, *Nutt.*
Spiranthes cernua, *Rich.*
tortilis, *Rich.*
Triphora pendula, *Nutt.*
Pogonia divaricata, *Nutt.*
ophioglossoides, *L.*
verticillata, *Muhl.*
Calopogon pulchellum, *Nutt.*
Corallorhiza multiflora, *Nutt.*
odonthorhiza, *Nutt.*
Epidendrum conopseum, *Ait.*
Bletia aphylla, *Nutt.*
verucunda, *Swartz.*
Tipularia discolor, *Nutt.*
Microstylis ophioglossoides, *Nutt.*
Orchis Elliottii, *Beck.*
nivea, *Nutt.*
Habenaria herbiola, *Br.*
ciliaris, *Br.*
cristata, *Br.*
blephariglottis, *Hook.*
Michauxii, *Nutt.*
repens, *Nutt.*

BROMELIACEÆ.

Tillandsia usneoides, *Pers.*
Agave Virginica, *L.*

AMARYLLIDACEÆ.

Amaryllis Atamasco, *L.*
Pancratium Mexicanum, *L.*

HÆMODORACEÆ.

Lachnanthes tinctoria, *Ell.*

IRIDACEÆ.

Iris hexagona, *Walt.*
versicolor, *L.*
tripetala, *Walt.*
Sisyrhinchium anceps, *L.*

DIOSCEREACEÆ.

Dioscorea quarternata, *Walt.*

SMILACEÆ.

Smilax hastata, *Willd.*
herbacea, *L.*
lanceolata, *Walt.*
laurifolia, *Walt.*
pumila, *Walt.*
rotundifolia, *L.*
glauca, *Walt.*

Medeola Virginica, *L.*
Trillium sessile, *L.*
Convallaria multiflora.
Uvularia perfoliata, *L.*
sessilifolia, *L.*

LILIACEÆ.

Lilium Catesbæi, *Walt.*
Carolinianum, *Mx.*
Yucca fiilamentosa, *L.*
gloriosa, *L.*
Erythronium Americanum, *L.*
Allium striatum, *Pursh.*
mutabile, *Mx.*
Aletris farinosa, *L.*
aurea, *Walt.*

PONTEDERIACEÆ.

Pontederia cordata, *L.*
Syena fluviatilis.

MELANTHACEÆ.

Zigadenus glaberrimus, *Mx.*
Leimanthium Virginicum, *Mx.*
Veratrum angustifolium, *Pursh.*
floccosum, sp. nov.
Tofieldia pubens, *Dryand.*
Amianthium muscætoxicum, *G.*
angustifolium, *Gray.*
Helonias dioica, *Pursh.*

JUNCACEÆ.

Juncus biflorus, *Ell.*
repens, *Mx.*
echinatus, *Muhl.*
filiformis, *Ell. Mx?*
effusus, *L.*
acutus, *L.*
polycephalus, *Mx.*
tenuis, *Willd.*
dichotomus, *Ell.*
aristulatus, *Mx.*
erythrocarpus, sp. nov?
Luzula campestris, *D.C.*
Pleea tenuifolia, *Mx.*

COMMELYNACEÆ.

Commelyna erecta, *Willd.*
Virginica, *L.*
communis.
Tradescantia Virginica, *L.*

XYRIDACEÆ.

Xyris fimbriata, *Ell.*
juncea, *Baldwin.*
flexuosa, *Ell.*
brevifolia, *Mx.*

ERIOCAULONACEÆ.

Eriocaulon floridulum, *Mx.*
villosum, *Walt.*
gnaphaloides, *Mx.*
decangulare, *Mx.*

CYPERACEÆ.

Dulichium spathaceum, *Pers.*
Kyllingia sesquiflora, *Torr.*
pumila.
Lipocarpha maculata, *Torr.*
Mariscus retroflexus, *Vahl.*
Trichelostylis mucronulata, *T.*
Cyperus Baldwiniana, *Torr.*
compressus, *L.*
flavescens, *L.*
Gatesii, *T.*
leptos, *Schultes.*
ovularis, *T.*
virens, *Mx.*
strigosus, *L.*
vegetus, *Willd.*
repens, *Ell.*
Nuttallii, *T.*
tetragonus, *Ell.*
speciosus, *Vahl.*
erythrorhizos.
Drummondii, *T.*
Eleocharis acicularis, *Br.*
capitata, *Br.*
melanocarpa, *T.*
microcarpa, *T.*
tricostata, *T.*
prolifera, *T.*
Robbinsii, *Oakes.?*
tuberculosa, *Mx.*
obtusa, *Schultes.*
equisetoides, *Ell.*
intermedia? *T.*
uncialis, sp. nov.
Scripus divaricatus, *Ell.*
Eriophorum, *L.*
Scripus lacustris, *L.*
triqueter, *L.*
Fuirena squarrosa, *Mx.*
scirpoidea, *Mx.*
Fimbristylis congesta, *T.*
spadicea, *Vahl.*
Baldwinii, *T.*
Isolepis Warei, *T.*
capillaris, *T.*
ciliatifolia, *T.*
coarctata, *T.*
Ceratoschœnus corniculatus, *G.*
Cephaloschœnus polystachys.
sp. nov.
Psilocarya rhynchosporoides, *T.*
Dichromena latifolia, *Bald.*
leucocephala.
Schœnus nigricans, *L.*
Cladium effusum, *T.*
Hypoporum gracile, *T.*
Baldwinii, *T.*
interruptum, *T.*
verticillatum.
Scleria oligantha, *Mx.*
triglomerata, *Mx.*
reticularis, *Mx.*
Caroliniana, *Willd.*
laxa, *T.*
pauciflora, *Muhl.*
Rhyncospora Baldwinii, *Gray.*
caduca, *Ell.*
cephalantha, *Gray*
ciliata, *Vahl.*
cymosa, *Nutt.*
Elliottii, *Gray.*
filifolia, *Gray.*
fascicularis, *Nutt.*
gracilenta, *Gray.*
megalocarpa, *G.*

Rhyncospora microcarpa, *Bald.*
miliacea, *Gray.*
multiflora, *Gray.*
oligantha, *Gray.*
plumosa, *Ell.*
paniculata, *Gray.*
rariflora, *Ell.*
distans, *Nutt.*
?pusilla, sp. nov.
?Grayana, sp. nov.
glomerata, *Vahl.*

Carex anceps, *Muhl.*
cephalophora, *Muhl.*
Cherokeensis, *Schw.*
dasycarpa, *Muhl.*
Elliottii, *Schw. & Tor.*
festucacea, *Schr.*
debilis, *Mx.*
Floridana, *Schw.*
foliculata, *L.*
glaucescens, *Ell.*
hirsuta, *Willd.*
multiflora, *Muhl.*
laxiflora? *Lam.*
digitalis, *Willd.*
polytrichoides, *Muhl.*
rosea.
stellulata, *Good.*
venusta? *Dew.*
tentaculata, *Muhl.*
lupulina, b. *Schw.*
Willdenovii, *Muhl.*
Baltzellii, sp. nov.

GRAMINEÆ.

Panicum Alabamense, *Nees.*
capillare, *L.*
Panicum angustifolium, *Ell.*
anceps, *Mx.*
Crus-galli, *L.*
debile, *Ell.*
dichotomum, *L.*
gibbum, *Ell.*
hians, *Ell.*
latifolium, *L.*
microcarpum, *Muhl.*
gymnocarpum, *Ell.*
multiflorum, *Ell.*
nitidum, *Lam.*
scabriusculum, *Ell.*
virgatum, *L.*
amarum, *Ell.*
Walteri, *Ell.*
geniculatum, *Muhl.*

Digitaria filiformis, *Ell.*
sanguinalis, *Scop.*

Setaria glauca, *P. de B.*

Paspalum Floridanum,
ciliatifolium, *Mx.*
distichum, *L.*
præcox, *Walt.*
purpurascens, *Ell.*

Ceresia fluitans, *Ell.*
sp. nov.

Aulaxanthus ciliatus, *Ell.*
rufus, *Ell.*

Orthopogon hirtellum, *Nutt.*

Cenchrus echinatus, *Willd.*

Tripsacum dactyloides, *L.*

Trisetum palustre.

Stipa avenacea, *L.*

Aristida lanosa, *Ell.*
spiciformis, *Ell.*
stricta, *Mx.*
purpurascens.

Festuca nutans, *Willd.*

Festuca tenella, *Ell.*
myurus.
Kœleria truncata, *Torr.*
Melica speciosa, *Muhl.*
Chloris petræa, *Mx.*
Monocera aromatica, *Ell.*
Poa ambigua, *Ell.*
annua, *L.*
autumnalis, *Muhl.*
capillaris, *L.*
Eragrostis, *L.*
hirsuta, *Mx.*
nitida, *Ell.*
nervata.
reptans, *Mx.*
pectinata, *Mx.*
refracta. *Muhl.*
conferta, *Ell.*
sesslerioides.
Uniola gracilis, *Mx.*
latifolia, *Mx.*
nitida, *Bald.*
paniculata, *L.*
Cynodon dactylon, *Pers.*
Eleusine Indica, *Lam.*
cruciata, *Ell.*
Elymus, sp.
Rottbœllia cylindrica.
ciliata, *Nutt.*
rugosa, *Nutt.*
Spartina glabra, *Muhl.*
juncea, *Willd.*
Zizania miliacea, *Mx.*
Gymnopogon racemosum.
Erianthus contortus, *Bald.*
strictus, *Bald.*
brevibarbis, *Bald.*
alopecuroides, *L.*
Leersia hexandra, *Kunth.*
Leersia lenticularis, *Ell.*
oryzoides, *L.*
Virginica, *L.*
Miegia gigantea, *Nutt.*
macrosperma, *Mx.*
Andropogon ciliatus, *Ell.*
furcatus, *Muhl.*
macrourus, *Mx.*
Neesii, *Trin.*
scoparius, *Mx.*
vaginatus, *Ell.*
melanocarpus, *Ell.*
tetrastachyus, *Ell.*
argenteus, *Ell.*

FILICES.

Aspidium acrostichoides, *Willd.*
Asplenoides, *Willd.*
cristatum, *Willd.*
thelypteris? *Willd.*
Asplenium ebeneum.
sp. nov.
Botrychium fumarioides, *Willd.*
Virginicum, *Swartz.*
Adiantum tenerum, *Willd.*
Polypodium incanum, *Swartz.*
hexagonopterum, *Mx.*
Woodwardia angustifolia, *Muhl.*
Virginica, *Willd.*
Onoclea sensibilis, *L.*
Pteris aqualina, *L.*
falcata, sp. nov.
Osmunda Cinnamonea, *L.*
regalis, *Mx.*
Lycopodium alopecuroides, *L.*
Carolinianum, *Mx.*
rupestre? *L.*
albidulum, *Mx.*

